# Neuronal Glycoprotein M6a: An Emerging Molecule in Chemical Synapse Formation and Dysfunction

**DOI:** 10.3389/fnsyn.2021.661681

**Published:** 2021-05-04

**Authors:** Antonella León, Gabriela I. Aparicio, Camila Scorticati

**Affiliations:** Instituto de Investigaciones Biotecnológicas “Rodolfo A. Ugalde”, Universidad Nacional de San Martín and Consejo Nacional de Investigaciones Científicas y Técnicas (IIBio-UNSAM-CONICET), Buenos Aires, Argentina

**Keywords:** GPM6A, neuronal plasticity, synaptopathy, synaptic interactome, PLP family

## Abstract

The cellular and molecular mechanisms underlying neuropsychiatric and neurodevelopmental disorders show that most of them can be categorized as synaptopathies—or damage of synaptic function and plasticity. Synaptic formation and maintenance are orchestrated by protein complexes that are in turn regulated in space and time during neuronal development allowing synaptic plasticity. However, the exact mechanisms by which these processes are managed remain unknown. Large-scale genomic and proteomic projects led to the discovery of new molecules and their associated variants as disease risk factors. Neuronal glycoprotein M6a, encoded by the *GPM6A* gene is emerging as one of these molecules. M6a has been involved in neuron development and synapse formation and plasticity, and was also recently proposed as a gene-target in various neuropsychiatric disorders where it could also be used as a biomarker. In this review, we provide an overview of the structure and molecular mechanisms by which glycoprotein M6a participates in synapse formation and maintenance. We also review evidence collected from patients carrying mutations in the *GPM6A* gene; animal models, and *in vitro* studies that together emphasize the relevance of M6a, particularly in synapses and in neurological conditions.

## Introduction

The excitatory synapses are specific neuron–neuron communications between axon and dendritic processes that orchestrate the information stream and storage in the brain (Tu et al., 2018). Synapse formation involves a complex series of events with at least three primary stages: axon elongation and guidance by which axons reach their target area; synaptic specificity governed by an appropriate association of synaptic molecules, and synaptogenesis which creates functional synapses (Shen and Cowan, 2010).

Functional synapses are supported by specialized protein complexes, whose function is regulated in time and space during neuronal development, allowing effective synaptic plasticity (Torres et al., 2017). The cellular and molecular mechanisms underlying neuropsychiatric and neurodegenerative disorders reveal that most of them can be classified as synaptopathies (Sengpiel, 2018), however, their full understanding so far remains unknown. Large scale genome-wide association studies (GWAS, reviewed in Claussnitzer et al., 2020) promote the study of additional candidates. Candidates arise either as genetic vulnerability or susceptibility loci allowing researchers to explore how they might be involved in the molecular mechanisms governing neurological diseases with complex etiology and heterogeneous genetic predisposition.

Neuronal glycoprotein M6a, encoded by the *GPM6A* gene, is a member of the tetraspan proteolipid protein (PLP) family together with PLP/DM20 and M6b. Since its discovery in 1992 (Baumrind et al., 1992; Lagenaur et al., 1992), M6a has emerged as one of many proteins involved in neuron development, synapse plasticity, and as a key component in various neuropsychiatric disorders (Michibata et al., 2009; El-Kordi et al., 2013; Gregor et al., 2014; Fuchsova et al., 2015). This review provides a quick overview of the structure and molecular mechanisms by which M6a participates in synapse formation and maintenance. Moreover, we review evidence collected from patients carrying mutations within *GPM6A*; animal models, and *in vitro* studies that highlight the relevance of M6a, particularly in synapses and related neurological conditions.

## Gene, Protein, and Structural Domains

**FIGURE 1:**
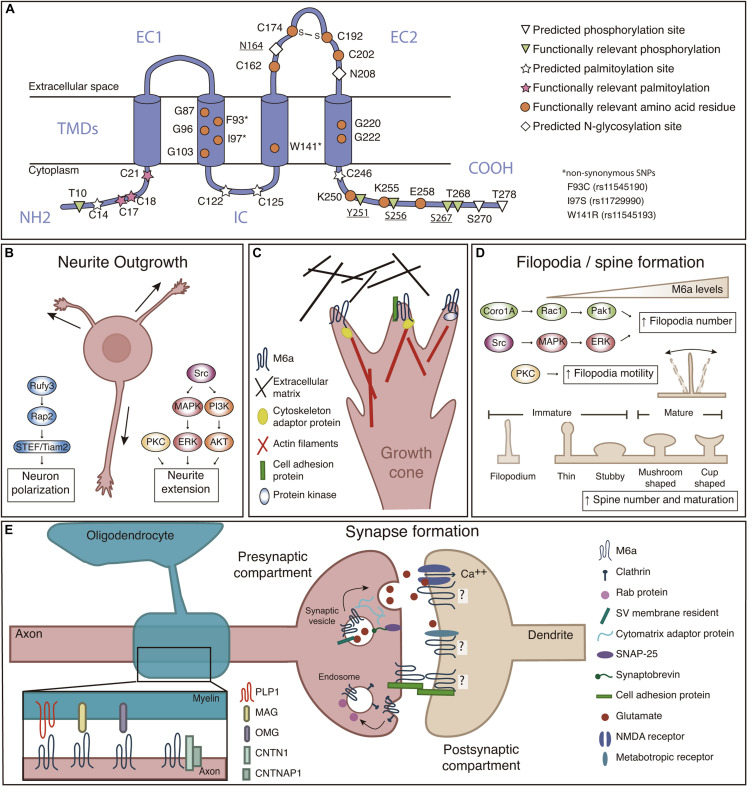
**(A)** M6a structural features. M6a’s topological computational model predicts four transmembrane domains (TMDs), two extracellular loops (a small one—EC1- and a large one—EC2-), an intracellular loop (IC), and N- and C-terminal ends facing the cell cytoplasm (NH2 and COOH). Predicted sites for palmitoylation (C14, C17, C18, C21, C122, C125, and C246), N-glycosylation (N164 and N208), and phosphorylation (T10, Y251, S256, S267, T268, S270, and T278) are highlighted; bold underlined sites indicate post-translational modifications which are experimentally validated. Orange circles represent amino acid residues relevant for M6a function. **(B)** M6a promotes neurite outgrowth and neuronal polarization, which involves the activation of Src/MAPK/ERK, PKC and PI3K/AKT, and Rufy3-Rap2-STEF/Tiam2 signaling pathways, respectively. **(C)** We hypothesize that M6a function depends on its association with partner proteins in specific membrane microdomains. These associations, induce auto/phosphorylation in specific C-terminal residues facing the cytoplasm, and finally promote neuronal plasticity (Fuchsova et al., 2009; Scorticati et al., 2011; Formoso et al., 2015a,b; Garcia et al., 2017; Honda et al., 2017; Aparicio et al., 2020). For example, M6a can associate with extracellular matrix proteins such as brevican and tenascin C through its extracellular loops in *trans* and/or with cell adhesion proteins (NCAM and NPTN) through its extracellular domains in *cis*, triggering its phosphorylation and therefore the recruitment of adapter proteins (such as Rap) and the activation of protein kinases that finally promote the reorganization of the cytoskeleton. **(D)** M6a promotes filopodia formation and motility through a mechanism that involves the activation of the Coro1A/Rac1/Pak1 and Src/MAPK/ERK, and PKC pathways, respectively. M6a also participates in spine formation and maturation of dendritic protrusions. **(E)** Left: Recently proposed interactions between M6a and myelin sheath proteins (PLP1, MAG, and OMG), and myelinated-axon proteins CNTN1 and CNTNAP1. Right: M6a is located at the presynaptic compartment plasma membrane, synaptic vesicle—SV—and endosomal membranes. M6a also interacts with postsynaptic glutamate receptors (like NMDA-R1 and GRM1) but its localization to the postsynaptic compartment was not experimentally confirmed yet. M6a, glycoprotein M6a; Src, Src kinase; MAPK, mitogen-activated protein kinase; ERK, extracellular signal-regulated kinase; PKC, protein kinase C; PI3K, phosphatidylinositide 3-kinase; AKT, serine/threonine-protein kinase; Rap2, Ras-related protein-2; Rufy3, Rufy 3 protein; STEF/Tiam2, T-lymphoma invasion and metastasis-inducing protein 2; NCAM, Neural cell adhesion molecule; NPTN, neuroplastin; Coro1A, coronin-1A; Rac1, Ras-related C3 botulinum toxin substrate 1; Pak1, p21-activated kinase 1; PLP1, proteolipid protein 1; MAG, myelin-associated glycoprotein; OMG, oligodendrocyte-myelin glycoprotein; CNTN1, contactin-1; CNTNAP1, contactin-associated protein 1; NMDA-R1, N-methyl-D-aspartate receptor 1; GRM1, metabotropic glutamate receptor 1; SNAP25, Synaptosomal-Associated Protein, 25 kDa.

The PLP family members are integral membrane proteins with a conserved topology: four transmembrane domains (TMDs), two extracellular loops (EC1 and EC2), one intracellular loop (IC), and the N- and C-termini both at the cytoplasmic face (Figure 1A). M6a exhibits low sequence identity with both PLP (38%) and M6b (52%), however, the TMDs are highly conserved (Greer and Lees, 2002; Fernandez et al., 2010).

Human glycoprotein M6a is encoded by a 369,731 kb gene organized into seven exons and located at chromosome 4q34.2. The full-length gene encodes a 278 amino acid membrane protein with a molecular mass of approximately 32 kDa (Olinsky et al., 1996). The amino acid sequence of M6a is highly conserved within mammals (more than 98% of identity). Post-translational modifications in M6a are summarized in Figure 1A. M6a has seven potential phosphorylation sites, and some of them are responsible for specific features described below. M6a has four cysteine residues within its EC2, critical for its folding and function, particularly C174 and C192 are linked by a disulfide bond, forming an intradomain important for protein-protein interactions (Fuchsova et al., 2009). The EC2 also contains two predicted N-glycosylation sites. Only glycosylation at N164 was experimentally corroborated, although there are no reported functions to date (Fang et al., 2016). Seven other cysteine residues close to the TMDs in the cytoplasmic side are potential palmitoylation sites, three of which (C17/18/21)—conserved within the PLP family—are necessary for M6a inclusion in lipid rafts (Honda et al., 2017; Ito et al., 2018).

GPM6A’s RNA-expression rapidly increases during development in human and murine brains. M6a is a brain-specific gene with a very high level of expression, representing one of the most abundant palmitoylated proteins in the CNS (Huminiecki et al., 2003; Kang et al., 2008). By contrast, low expression was detected in the lung, spleen, ovary, and the thyroid gland (Fagerberg et al., 2014; Yu et al., 2014; Yue et al., 2014). M6a protein levels are enriched in the hippocampus, cerebellum, striatum, and prefrontal cortex among other brain areas^1^. Regarding specific cell expression at the CNS, M6a is mostly placed at the cell surface of neurons and epithelial cells of the choroid plexus, but not in glial cells. The neuronal expression of M6a gives it a distinctive feature within the PLP family members as PLP is expressed only in glial cells and M6b is expressed in both neurons and glia (Werner et al., 2013).

## M6a and Synapse Formation

### M6a’s Role in the Presynaptic Formation

The coordination of neuronal differentiation, axonal growth, and guidance involves timely expression of cell surface proteins and extracellular adhesion molecules escorted by structural changes in the cellular cytoskeleton (Caceres et al., 2012). During neurite outgrowth, plasma membrane proteins are directed toward neurites first, and then they are concentrated in growth cones (GCs). There, proteins will be available to respond to orientation signals and to signal the path to a specific destination (Fuentes and Arregui, 2009; Ulloa et al., 2018). Indeed, M6a was identified as an “edge-membrane antigen” (EMA) because it was found concentrated at the edge of neuronal GCs and their lamellipodia in cultured neurons from the cerebellum, cortex, and hippocampus (Baumrind et al., 1992; Lagenaur et al., 1992). Later on, M6a was found to be critical for neurite growth in a wide variety of *in vitro* models, from brain tissue explants to neuronal cell lines and from human to Xenopus (Lagenaur et al., 1992; Mukobata et al., 2002; Alfonso et al., 2005; Zhao et al., 2008; Michibata et al., 2009; Formoso et al., 2015a). Noteworthy, Sato et al. (2011a) observed a reduction of the axon projections in the olfactory bulb from embryonic brains at E14.5 of Gpm6a knockout mice. Besides, double knockout mice for Gpm6a and Gpm6b show decreased axon elongation and a thinner corpus callosum which could be rescued by forced expression of M6 proteins (Mita et al., 2015).

To date, redundant signaling pathways of Src/MAPK/ERK, PKC, and PI3K/AKT kinases mediate M6a-induced neurite extension in hippocampal neurons and neuroblastoma cells (Mukobata et al., 2002; Scorticati et al., 2011; Formoso et al., 2015a). The phosphorylation of tyrosine 251 close to M6a’s C-terminus is critical for neuritogenesis (Formoso et al., 2015a). By contrast, cells expressing a truncated form of M6a lacking the N-terminus did not show neurite outgrowth inhibition (Mita et al., 2015). The direct association of M6a’s C-terminus with coronin1A—a cytoskeleton adaptor molecule—verify its essential role in the extension of projections (Alvarez Julia et al., 2016; Martorella et al., 2017). Furthermore, Honda et al. (2017) identified that M6a induces neuron polarity through the Rufy3-Rap2-STEF/Tiam2 pathway.

Recent proteomic studies identified proteins associated with M6a that participate in neurite outgrowth and axon elongation (Figures 1B,C; Sato et al., 2011a; Huttlin et al., 2017; Aparicio et al., 2020; Haenig et al., 2020; Pourhaghighi et al., 2020). To emphasize some examples, we divided them into 3 groups: (i) Cytoplasmic proteins: which include cytoskeleton adaptor molecules and cytoskeleton organization pathways which might interact with M6a’s cytoplasmic tails as Ras kinases 2, DiRas2 (Grunewald et al., 2021) and the kinases mentioned above. (ii) Cell adhesion molecules (CAMs): which mediate recognition of cellular targets, bind the cell surface to the extracellular matrix (ECM), and maintain cell shape. Examples of these molecules that could interact with M6a’s extracellular loops or its transmembrane domains are neuronal cell adhesion molecule (NCAM) (Neugebauer et al., 1988), and neuroplastin, NPTN (Beesley et al., 2014). (iii) ECM proteins: which only could interact with M6a’s extracellular loops: brevican (BCAN) (Miura et al., 2001), and tenascin C (TNC) (Andrews et al., 2009).

Presynaptic boutons are terminal specializations of the axon, which contain synaptic vesicles (SVs) filled with neurotransmitters. This specific compartmentalization involves the coordination of both SVs and presynaptic active zone proteins, which define regions in the membrane for the release of neurotransmitters to the synaptic cleft (Yue et al., 2014). Thus, there are three main types of proteins (i) residents of SV’s membrane, such as synaptophysin (Egbujo et al., 2016), (ii) filament or adaptor proteins of the cytomatrix, such as piccolo and bassoon (Hida and Ohtsuka, 2010), and (iii) those that participate in synaptic vesicle exocytosis such as synaptosomal associated protein 25 (SNAP25) (Antonucci et al., 2016; Figure 1E). Roussel et al. (1998) revealed that M6a was distributed at the presynaptic membrane, in particular, on the membrane of SVs which was confirmed by proteomic analysis (Takamori et al., 2006; Taoufiq et al., 2020). Besides, M6a was also found at glutamatergic nerve terminals in the cerebellum and cerebral cortex but not in GABAergic neurons of adult brain mice. This specific-excitatory preference was also confirmed in the hippocampal formation in which M6a colocalizes with the vesicular glutamate transporter VGLUT (Cooper et al., 2008).

Recently, M6a has also been associated with other SVs and presynaptic active zone—PAZ—residents proteins, like synaptic vesicle protein 2B (SV2B), piccolo, bassoon, and synapsin 1 (Aparicio et al., 2020). Overexpression of M6a in hippocampal neurons showed a significant increment of synaptophysin puncta correlating with an increase in the number of synapses. On the contrary, neurons subjected to siRNA depletion of M6a and neurons overexpressing a truncated form of M6a at the EC2 loop exhibited a decreased number of synaptophysin puncta (Alfonso et al., 2005; Fuchsova et al., 2009; Formoso et al., 2016). Moreover, M6a internalizes and recycles back to the cell membrane via clathrin-mediated endocytosis, and localizes in Rab5, Rab7, and Rab11 positive endosomes, just like the case of SVs recycling pathways (Wu et al., 2007; Formoso et al., 2016; Garcia et al., 2017; Rosas et al., 2018). Indeed, inducing M6a acute internalization correlated with a decrease of both the number of synaptophysin puncta and synapses (Garcia et al., 2017), suggesting that M6a might be playing an active role in SV/neurotransmitter release.

### M6a’s Role in the Postsynaptic Formation

Straight opposed to the presynaptic terminal is the postsynaptic target. Dendritic spines, the main postsynaptic compartment, are protrusions from the dendrite shaft that receive the information from axonal terminals through different neurotransmitter receptors. Dendritic spines can show dynamic changes in number, size, shape, and movement which allow synaptic rearrangements to take place (Tonnesen and Nagerl, 2016). This plastic feature is a critical substrate for functional plasticity during pruning or learning and memory, and also for synaptic dysfunction as observed in neurodegenerative and neuropsychiatric conditions including Alzheimer’s Disease (AD), autism spectral disorders (ASD), schizophrenia, and depression (Penzes et al., 2011; Bian et al., 2015; Ozcan, 2017).

According to its size and shape, dendritic spines can be classified as thin, stubby, mushroom, and cup-shaped. Moreover, according to their functionality, dendritic protussions can also be classified as immature (filopodia, thin and stubby) or mature (mushroom and cup-shaped) (Figure 1D). Three possible models have been proposed to explain how a synapse is formed: (i) Sotelo’s model describes that a synapse could arise when an immature spine is contacted by the axon terminal inducing its development toward the mushroom type (Sotelo et al., 1975), (ii) Miller/Peters’s model proposes that a presynaptic terminal directly contacts with the dendrite shaft, inducing spine outgrowth (Miller and Peters, 1981), and (iii) Filopodial model: where dendritic filopodia may actively initiate synaptogenic contacts by contacting a presynaptic terminal, thereby inducing its stabilization and subsequently maturation to the mushroom type (Yuste and Bonhoeffer, 2004; Ziv and Fisher-Lavie, 2014).

M6a has been widely described to be involved in filopodia/spine formation in different cell culture models (Alfonso et al., 2005; Sato et al., 2011b; Scorticati et al., 2011; Formoso et al., 2015b; Alvarez Julia et al., 2016; Formoso et al., 2016; Rosas et al., 2018). Two pathways have been implicated by which M6a increases filopodia density. M6a overexpression induces the activation by phosphorylation of the intracellular cascade involving the Src and MAPK/ERK pathway; and the localization of M6a within lipid rafts is compatible with this (Scorticati et al., 2011). Likewise, signaling pathways that include Rac1 and Pak1 activation through coronin1A facilitate M6a-induced filopodium formation (Alvarez Julia et al., 2016). There are key domains required for M6a to induce filopodia/spine formation (Figure 1D): (i) TMDs homotypic-interactions, especially commanded by particular glycine residues at TMD2 and TMD4 (Formoso et al., 2015b, 2016), (ii) a disulfide bridge at EC2 loop formed between cysteine residues C174 and C192 (Fuchsova et al., 2009), and (iii) C-terminal domain residues K250/K255/E258 (Rosas et al., 2018). Although the N-terminus is not involved in filopodia induction (Mita et al., 2015; Rosas et al., 2018), phosphorylation of certain serine/threonine residues at N-and C-termini, T10/S256/S267/T268, proved to be necessary for filopodial motility (Brocco et al., 2010).

Although M6a induces dendritic protrusions plasticity, whether or not it is placed at the postsynaptic membrane is undetermined. For instance, M6a was identified at presynaptic membranes and enriched in glutamatergic synaptic vesicles docked to the presynaptic active zone (Roussel et al., 1998; Boyken et al., 2013). Conversely, M6a was detected in a proteomic analysis of enriched postsynaptic membrane fractions from mice brains, suggesting a postsynaptic localization for M6a (Reim et al., 2017). Besides, we described that M6a co-immunoprecipitated with integral components of the postsynaptic membranes such as metabotropic glutamate receptor 1 (GRM1), voltage-dependent anion channel 1 (VDAC1), and N-methyl-D-aspartate receptor type 1, NMDA-R1 (GR1A1) (Aparicio et al., 2020). Indeed, M6a-overexpressing neurons exhibited an increase in the number of NMDA-R1 clusters, whilst truncated forms of M6a or its depletion decreased the number of NMDA-R1 clusters (Formoso et al., 2016; Garcia et al., 2017). The colocalization of M6a and NMDA-R1 suggests that M6a acts as a scaffold protein assembling proteins and lipids to form a signaling platform on the neuronal surface (Wu et al., 2007; Scorticati et al., 2011). However, to determine whether M6a is indeed located at the postsynaptic membrane techniques such as cryo-electron microscopy and super-resolution microscopy are needed to avoid contaminations of biochemical-based techniques (Liu et al., 2019; Nosov et al., 2020).

## M6a’s Potential Role in Neuron-Glia Interaction

M6a is enriched within cerebellar parallel and hippocampal mossy fibers, whose axons remain unmyelinated also in adulthood (Cooper et al., 2008). Nonetheless, new data suggest that M6a could participate in neuron-glia interactions. This subject exceeds the topics covered in this review, and no functional experiments were reported yet, but we would like to highlight a few insights. Pourhaghighi et al. (2020) identified associated protein complexes in adult mammal brains and built BraInMap^2^. M6a was found within a complex of 30 interacting proteins, some of which are myelin sheath proteins, including the main myelin glycoproteins PLP and MAG; or are myelin sheath associated proteins like contactin 1 and contactin associated protein 1. In agreement, we identified 20 myelin proteins in the co-immunoprecipitation complexes formed by M6a’s extracellular domains and rat hippocampal samples, in which PLP was experimentally confirmed (Aparicio et al., 2020). Also, Jahn et al. (2020) identified M6a in the myelin sheath of post mortem human brains.

## M6a in Synapse Function and Dysfunction

The evidence reviewed so far highlights an important role of M6a in neuronal development and synaptic plasticity. However, few reports interrogate about the role of M6a on active synapses. All of them are based on determining the cluster number of synaptophysin and/or the number of clusters between synaptophysin and NMDA-R1 on *in vitro* models (Alfonso et al., 2005; Fuchsova et al., 2009; Formoso et al., 2016; Garcia et al., 2017). For instance, endogenous M6a acute depletion using siRNA or treatments with M6a-mAb neutralizing antibodies dramatically decrease the number of synaptophysin clusters, or synaptophysin and NMDA-R1 colocalization clusters (Alfonso et al., 2005; Garcia et al., 2017). The latter suggests that M6a is not only required for the formation of synapses but also their maintenance. Evidence from M6a’s relevance *in vivo* comes from the observation that several *GPM6A* variants and inadequate expression levels were associated with several neuropsychiatric disorders (see Table 1), thus highlighting that the impairment of M6a function contributes to disease onset or increases the risk of susceptibility.

**TABLE 1:** Summary of *GPM6A* gene variants and expression levels related to disorders.

**Alteration**	**Species**	**Approach**	**Sample**	**Study**	**Result**	**References**
Chronic Stress	Rats	Restraint stress	HP	mRNA	↓	Alfonso et al., 2002
	Shrews	Psychosocial stress	HP	mRNA	↓	Alfonso et al., 2004a,b
	Mice	Restraint stress	HP	mRNA	↓	Alfonso et al., 2006
	Mice	Prenatal stress	HP	mRNA	↑	Monteleone et al., 2014
Claustrophobia	Mice	Gpm6a−/−		Behavioral test	↓	El-Kordi et al., 2013
	Humans			Genotyping	GPM6A variant 3‘UTR	
Neuroticism	Humans			GWAS	rs17611770	Nagel et al., 2018
Depression	Humans		HP/PFC	mRNA	↓	Fuchsova et al., 2015
	Humans		Saliva	ELISA	ND and ↑	Monteleone et al., 2020
Bipolar Disorder	Humans			GWAS	rs17599018	Greenwood et al., 2012
Schizophrenia	Humans	with depression		Genotyping	rs10520303	Boks et al., 2008
	Humans			GWAS	rs1106568, rs62334820, rs13142920, rs7673823, rs6846161	Schizophrenia Working Group of the Psychiatric Genomics Consortium, 2014; Li et al., 2017; Pardiñas et al., 2018; Lam et al., 2019
Learning disability	Humans			Genotyping Microarray	GPM6A duplication	Gregor et al., 2014
Alzheimer Disease	Mice	APP/PS1 mice	OB	Proteomics	↑	Lachén-Montes et al., 2016
	Humans		HP	mRNA microarray	↓	Xu et al., 2006
	Humans	EVs	Cortical gray matter	Proteomics	↑	Muraoka et al., 2020; Quiroz-Baez et al., 2020
	Humans	EVs	CSF	Proteomics	↑	Chiasserini et al., 2020
Autistic spectrum disorder	Mice	PSD membrane fractions	Striatum and HP	Proteomics	ND	Reim et al., 2017
	Humans		CB	Proteomics	↑	Abraham et al., 2019

Chronic stress induces behavioral changes in mammals, which can be evidenced by mood disorders including anxiety, claustrophobia, and depression. In this context mRNA levels of Gpm6a were decreased in the hippocampus of chronically stressed animals (Alfonso et al., 2002, 2004a,b); and then rescued with the administration of antidepressants (Alfonso et al., 2006). These are consistent with findings where chronically stress animals presented a reduction of the dendritic arborization and structural changes in the mossy fiber terminals (Cooper et al., 2008). In humans, *GPM6A* mRNA levels were also decreased in the hippocampus of depressed patients who committed suicide (Fuchsova et al., 2015). Furthermore, depressed patients treated with serotonin reuptake inhibitors showed a reduction of M6a levels in saliva compared to depressed patients under benzodiazepine treatment or no treatment at all. As a result, M6a was proposed as a mood disorder biomarker (Monteleone et al., 2020).

Gpm6a knockout mice lack obvious behavioral abnormalities but when subjected to moderate restrain stress, they presented a claustrophobia-like phenotype. These findings encouraged El-Kordi and collaborators to investigate *GPM6A* variants within claustrophobic patients. In their study they found a 3′-untranslated region variant that produced a functional mRNA that could not be silenced by neuronal miR124, thereby losing the physiological regulation of the gene (El-Kordi et al., 2013). Gregor et al. (2014) identified a *de novo* duplication of the *GPM6A* gene in a patient with learning disabilities and behavioral anomalies. Furthermore, Rao-Ruiz et al. (2015) revealed that in mice subjected to a contextual fear-memory learning task M6a was differentially up-regulated 4 h later. Until now, there was no direct relationship between M6a levels and the molecular processes underlying memory and learning formation. However, these independent findings suggest that this may warrant further investigation.

The intrinsic genetic program responsible for synapse formation and maintenance involves a correct pruning of the dendritic spines. An abnormal dendritic spine pruning during development and in adulthood contributes to the generation of synaptopathies such as ASD, schizophrenia, or AD (Penzes et al., 2011). For instance, subjects with ASD have deficits in social interactions, disruption of oral communication, and present repetitive behavior; correlated with an elevated number of dendritic spines and a late onset of the pruning process (Toro et al., 2010). Interestingly, a proteomic analysis done by Abraham et al. (2019) revealed that M6a levels were increased in samples from the cerebellum of patients with ASD. Moreover, proteomic studies showing altered M6a levels in both animal models and patients with AD contribute to a possible role in memory decline (Xu et al., 2006; Lachén-Montes et al., 2016; Chiasserini et al., 2020; Muraoka et al., 2020). Also, M6a is downregulated in the hippocampal formation whereas it is enriched in extracellular vesicles from cerebrospinal fluid (CSF) samples of AD patients suggesting an active secretion of M6a during AD progression (Xu et al., 2006; Chiasserini et al., 2020). Indeed, Muraoka et al. (2020) proposed M6a, together with ANX5, VGF, and ACTZ as a biomarker for monitoring AD progression from CSF samples (Muraoka et al., 2020).

One of the goals of genome-wide association studies is to link specific genetic variants with specific phenotypes. This information can then be used to evaluate the risk or susceptibility of a population to develop a certain disease, in diagnostic tests or to guide treatments. Indeed, single nucleotide polymorphisms (SNPs) within non-coding region of *GPM6A* have been linked to schizophrenia (Boks et al., 2008; Schizophrenia Working Group of the Psychiatric Genomics Consortium, 2014; Li et al., 2017; Pardiñas et al., 2018; Lam et al., 2019), and one variant for both neuroticism (Nagel et al., 2018) and bipolar disorder (Greenwood et al., 2012). However, there are still no reports confirming whether any of these SNPs lead to changes in M6a gene expression or function. Our lab studied 3 non-synonymous SNPs within *GPM6A*’*s* TMD coding region reported in the dbSNP database^3^. By doing reverse genetic experiments, we demonstrated that all nsSNPs prevented M6a from being functional in neurons, impaired formation of dendritic spines and synapses owing to decreased stability, dimerization, or improper folding of the protein (Formoso et al., 2015b, 2016).

## Concluding Remarks

Evidence collected over 30 years since its discovery show that glycoprotein M6a has a critical role in synapse formation, plasticity, and maintenance. Previous research so far has focused on *in vitro* approaches, with only a few articles studying M6a-deficient mice. Unfortunately, in none of those studies the synaptic activity or synaptic integrity were interrogated. New data coming from “omic” and GWAS approaches in combination with basic investigation will expand our knowledge of the field and define the exact role of *GPM6A* in neuronal development and synaptopathies. This in turn will offer new routes to improve diagnosis and develop more effective treatments.

## Author Contributions

AL, GA, and CS were involved in bibliography revision. CS contributed to the design and conceptualization of the research topic. CS, AL, and GA wrote the manuscript. AL and GA made the figure and the table. All authors contributed to the article and approved the submitted version.

## Conflict of Interest

The authors declare that the research was conducted in the absence of any commercial or financial relationships that could be construed as a potential conflict of interest.

## Footnotes

1 www.proteinatlas.org

2 www.bu.edu/dbin/cnsb/mousebrain

3 https://www.ncbi.nlm.nih.gov/snp/
